# Ab Initio Binocular Formulation of Listing’s Law

**DOI:** 10.3390/jemr19030056

**Published:** 2026-05-16

**Authors:** Jacek Turski

**Affiliations:** Department of Mathematics and Statistics, University of Houston-Downtown, One Main Street, Houston, TX 77002, USA; turskij@gmail.com

**Keywords:** eye’s misaligned optics, the asymmetric eye model, ocular torsion, Rodrigues’ vector, Listing’s law, half-angle rule, noncommutativity of eye rotations, configuration space of eye’s rotational sequences, *GeoGebra*’s dynamic geometry

## Abstract

Human eyes do not have perfectly aligned optical components; the fovea is displaced from the posterior pole, and the crystalline lens is tilted away from the eye’s optical axis. Important in the study of vision quality, it is included here in binocular and oculomotor research. In the binocular system, with the eye’s optical asymmetry, all axes differ. The eye’s posture change is decomposed into the torsion-free part that gives the change in visual axis direction and the torsional part that best approximates the rotation about the lens’s optical axis. This geometric formulation, supported by computer simulations and modern ophthalmology studies, leads to binocular Listing’s law and the related half-angle rule, important for oculomotor control by constraining the eye’s redundant torsional degree of freedom. The eye’s primary position and the Listing plane, indispensable ingredients of Listing’s law, are replaced with the binocular eyes’ posture corresponding to the eye muscles’ natural tonus resting position, which serves as a zero-reference level for convergence effort. Further, the binocular constraints couple 3D changes in the torsional positions of the eyes within the ab initio formulation of Listing’s law here, which was previously proposed ad hoc. Finally, the noncommutativity rule underlying Listing’s law and the half-angle rule are discussed by specifying the configuration space of sequences of fixations of binocularly constrained eyes, which are visualized in 3D simulations. The results obtained in this study should be a part of the answers to the questions posted in the literature on the relevance of Listing’s law to clinical practices.

## 1. Introduction

Listing’s law, as formulated by Hermann von Helmholtz [1], states that for a stationary, upright head from a reference position, the single eye rotation axes lie in a plane referred to as the displacement plane. The displacement planes associated with different reference positions do not coincide. When the reference position is perpendicular to the displacement plane, the plane is called the “Listing plane”, and the unique reference position is the “primary position”. Further, for any eye’s tertiary position, the displacement plane is obtained by rotating the Listing plane by the half-angle of gaze eccentricity. This important corollary of Listing’s law is known as the half-angle rule and is usually attributed to the noncommutativity of 3D rotations [2].

### 1.1. Shortcomings of the Standard Theories

Although Helmholtz provided appealing arguments in support of Listing’s law, which is now one of the best studied findings in oculomotor research [3,4,5,6,7,8,9,10,11,12,13,14], some shortcomings have been addressed in recent years. To review them, I first note that the eye movement recordings have shown that during convergence on a near object, the eyes’ rotation vectors remain confined to temporally rotated Listing’s planes by angles proportional to the vergence for the right and left eye [7,8,9,13]. This is referred to as L2 law, while the original, monocular Listing’s law is L1. Nevertheless, the experimental data in the recordings show that in L2 law, the coefficient of proportionality ranges from 0.2 to 0.5. Thus, L2 should be considered an ad hoc binocular extension of Listing’s law.

This lack of a consistent binocular formulation is evident in the commonly expressed view that Listing’s law constrains the redundant torsional degrees of freedom of a single eye to support neural processing in building our spatial understanding [1]. However, this goal cannot be fully met because we perceive scenes through bifoveal fixations on near objects, as we live in closed quarters for a significant portion of the time. Additionally, the findings in [15] support the idea that a binocular control system couples the 3D movements of the eyes and that an existing model of monocular torsion should be generalized to the binocular case.

Further, as discussed in [15], there is no convincing definition of the eye’s primary position and the Listing plane, both indispensable ingredients of Listing’s law. Consequently, as indicated in [16], the neurophysiological significance of the primary position and the Listing plane has remained elusive despite their importance in oculomotor control. Moreover, following [15], the older theory put forward by Fick and Wundt (see [1]) and the more modern mathematical formulation in [17] are not convincing. For example, whether Listing’s law is obeyed with the least effort is uncertain, as is the lack of explanation of the vergence-dependent departures from Listing’s law.

On the other hand, by discussing oculomotor abnormalities in neurological disorders and in strabismus and Listing’s law that is obeyed in non-primate animals like chameleons, [18,19] concluded that the functional meaning of Listing’s law is still incompletely understood.

Another shortcoming is failing to account for the ubiquitous misalignment of the human eye’s optical components; the fovea is displaced from the posterior pole by about 8° measured at the eye’s rotation center, and the crystalline lens is tilted away from the optical axis [20,21,22,23,24]. The fovea’s anatomical displacement, relatively stable in the human population, and the cornea’s asphericity contribute to optical aberrations, and the lens tilt is adjusted to partially compensate for these aberrations [25,26,27,28]. Thus, despite many definitions of the primary position and the Listing plane discussed in the literature [1,29,30,31], no one can be satisfied with the healthy human eye’s misaligned optical components.

### 1.2. Recent Developments

The effects of misaligned optical components in the eye on binocular vision have only recently been studied. In [32,33,34], based on the 2D asymmetric eye (AE) model developed by the author, the geometric study of binocular vision included the fovea’s horizontal displacement and the crystalline lens’ horizontal tilt. In [35], the vertical misalignment of the eye’s optics was added to the horizontal misalignment, resulting in the 3D AE. The results included the 3D iso-disparity curves (the horopter is a zero-disparity curve defined below) and an explanation of the subjective tilt of the vertical horopter, which agrees with recent experimental results in [36,37] but not with the data of Helmholtz and others (see discussion in [36]).

One important example in the research based on the AE model is the following. In binocular vision, retinal correspondence refers to a small retinal area in one eye and a corresponding unique area in the other that shares a single subjective visual direction. Furthermore, it explains the shape of the horopters, i.e., the zero-disparity locus of points in space, such that each point on the horopter projects to a pair of corresponding retinal elements. This correspondence organizes 3D perception, i.e., stereopsis and the geometry of visual space. The distribution of corresponding elements is asymmetric on the retina—the corresponding elements are compressed in the temporal retinae relative to those in the nasal retinae. It was demonstrated in [34] for the first time that this asymmetry is caused by the misalignment of the eye’s optical components, which explains the shape variation resembling conic sections of the empirical horopters rather than the commonly considered Vieth-Müller circle (VMC); see Section 2.2.

### 1.3. The Model Preceding AE

The AE model has a curious predecessor in the field of vision science. In 1959, Frederick Verhoeff wrote in [38]: “[…] because we see straight lines with spherical retinas, mathematically, the eyes can be replaced with ‘miniature pinhole cameras’.” He placed two cameras at the interocular distance, pointing straight forward, so their screens are in the same plane. Further, Verhoeff accounted for the alpha angle—the angular displacement of the fovea on the retina from the posterior pole, which he believed might benefit binocular vision. To do this, he first assumed that the distance and direction of the corresponding points from the foveae (the optical centers) were the same on both camera screens. Then he moved the principal points, i.e., the foveae, on the screen of the right camera to the right and on the left camera to the left, so that the cameras would have equal non-zero angles alpha. The Verhoeff eye model corresponds to the 2D AE with the fovea displaced by angle alpha, but does not include a tilted lens or angle beta discussed in Section 2.2.

Interestingly, three years after Verhoeff introduced his eye model of the flat-screen pinhole camera with angle alpha, he used it in [39] to explain Kundt and Tschermak-Seysenegg’s illusions.

### 1.4. This Study: Binocular Listing’s Law for AEs

The presented article focuses on Listing’s law. The inclusion of misalignment in the optical components of the eyes in their study will enable definitive, ab initio binocular formulation within the framework of the 3D AE model. I should stress that Listing’s law, as formulated by Helmholtz and discussed above, restricts Donders’ law, which is a general statement for a single eye, stating that with the head erect and looking at infinity, any gaze direction has a unique torsional angle, regardless of the path the eye follows to get there.

Donders’ law arises from the organization of neural circuits for eye movements: Neurons in areas such as the superior colliculus (SC), cerebellum, and brainstem oculomotor nuclei encode eye position and movement commands. These signals are structured such that the torsional components of the eye position are uniquely constrained by the 2D gaze direction, encoded by the population of neurons via each neuron’s “preferred direction”. The cerebellum is crucial for maintaining Donders’ law; if disrupted, eye movements can violate it. The implementation of Donders’ law is performed by the brainstem: neurons in the oculomotor nucleus send signals to the eye muscles, which are already constrained by upstream processing, so that the muscles themselves do not enforce Donders’ law; the neural commands do. In experiments with monkeys, researchers record from neurons in areas like the SC, brainstem burst neurons, and cerebellar regions. They found that many neurons encode 2D gaze direction with little or no independent encoding of torsion, consistent with Donders’ law. Some of these aspects are discussed in Section 5.

However, it is unclear how the binocular Listing’s law formulated in this study relates to the monocular Donders’ law, the neural basis reviewed above. As mentioned in [15], a binocular control system couples the 3D movements of the eyes, and an existing model of monocular torsion should be generalized to the binocular case.

The binocular Listing’s law and the related half-angle rule formulated here are supported by ophthalmology and experimental studies and are confirmed by new geometric formulations and numerical simulations. The unique results include a first full description of the configuration space of sequences of binocularly constrained eyes’ bifixations underlying Listing’s law, including the half-angle rule when bifixating eyes change between tertiary positions, and the underlying noncommutativity. Table 1 summarizes the notation and important geometrical facts used in this paper.

**Figure 1:**
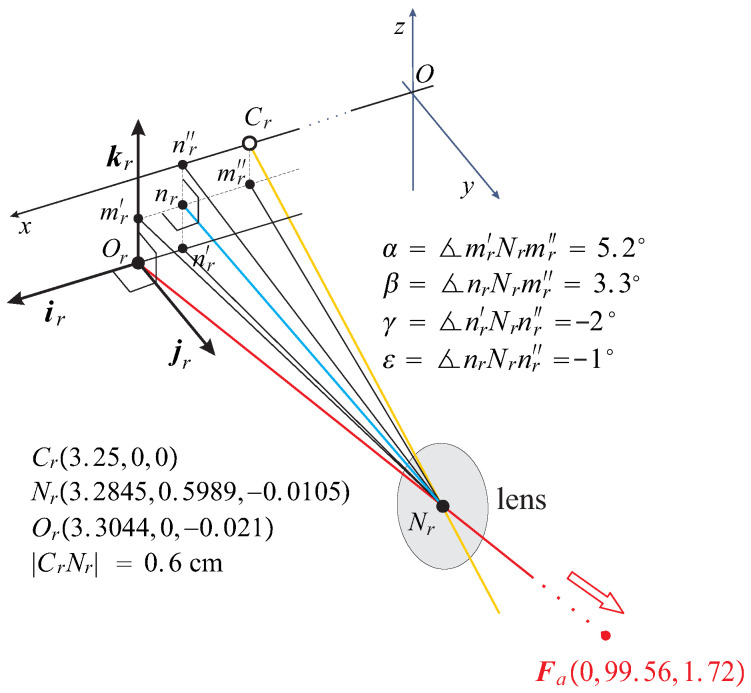
The right AE view is shown in the ERP near the optical center $O_{r}$ and the eye’s rotation center $C_{r}$, both in the xz-plane of the head’s fronto-parallel plane. $N_{r}$ is the nodal point; the yellow line is the eye’s optical axis passing through $N_{r}$ and $C_{r}$; and the red line is the visual axis passing through fixation point $F_{a}$ and $O_{r}$ and hence also passing through the fovea. Finally, the blue line through $N_{r}$ and its perpendicular projection into the image plane (fronto-parallel plane in ERP) is the lens’s optical axis. The typical values of misalignment angles in the human population determine the ERP fixation at $F_{a}\left(0,99.56,1.72\right)$. The AE’s image plane orientation is specified by the frame $\left(i_{r},j_{r},k_{r}\right)$ attached at the optical center $O_{r}$, the fovea’s projection along the visual axis into the image plane. The left eye in this posture is mirror-symmetric in the yz-plane. Note that all axes of the eye differ.

**Figure 2:**
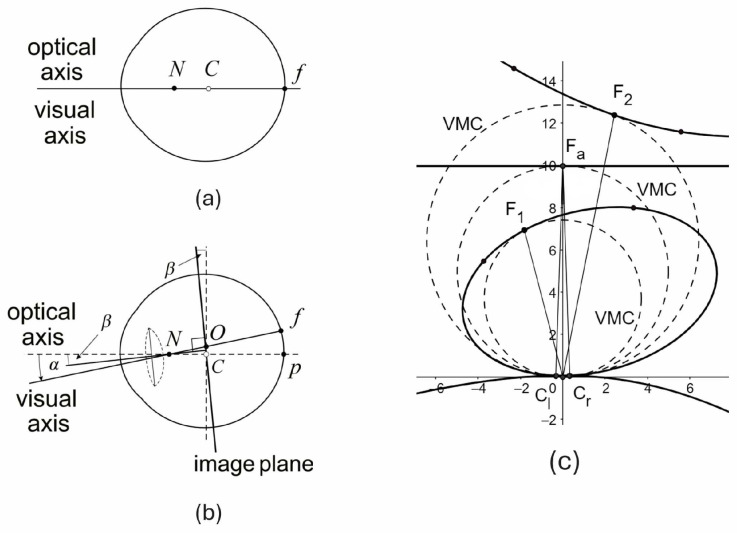
2D AE vs SE in binocular vision. (**a**) The standard reduced eye model—the SE model. (**b**) The right AE model. *N* is the nodal point, *C* is the eye’s rotation center, *f* is the fovea, and *p* is the posterior pole. The angles α and β in the AE model represent the horizontal misalignment of the fovea and the lens, respectively. The image plane in the AE is parallel to the lens’s equatorial plane and passes through the eye’s rotation center. *O* is the projection of the fovea into the image plane—the optical center on the image plane. (**c**) Horopteric conics, closely resembling empirical horopters, were obtained in the binocular system, with AEs shown as continuous lines, for anthropomorphic dimensions and for typical misalignment angles α=5.2° and β=3.3° in the human population. The VMCs shown in dashed lines are the geometric horopters for the SE. Although they approximate the empirical horopters well near fixation, they differ substantially in the periphery, which affects our sense of immersion in the 3D environment despite receiving 2D projections.

**Figure 3:**
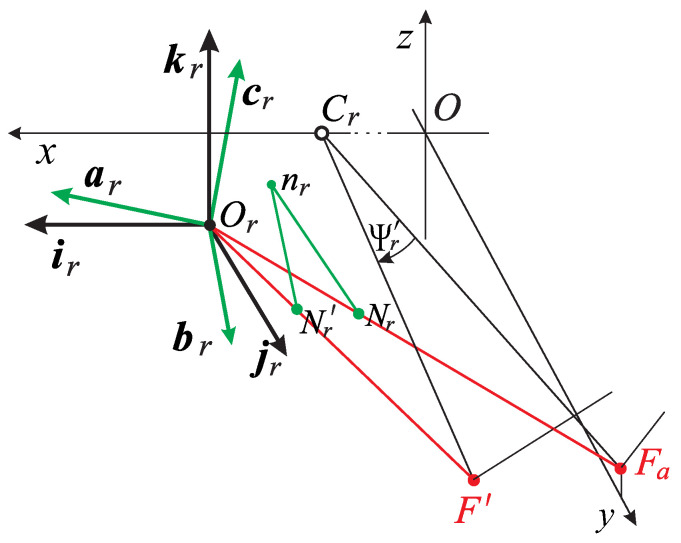
In the ERP, the image plane of each AE agrees with the head frontal xz-plane. The black frame $\left(i_{r},j_{r},k_{r}\right)$ attached at optical center $O_{r}$ of the image plane, the visual axis (the red line through $O_{r}$ and $F_{a}$), and the lens’ optical axis (the green line segment between $n_{r}$ and the nodal point $N_{r}$) are rigidly rotating with the image plane when the eyes’ rotation changes the fixation axis through $F_{a}$ into a fixation axis through rotated nodal point $N_{r}^{\prime }$ and $F^{\prime }$. The rigidly green frame $\left(a_{r},b_{r},c_{r}\right)$, the rigidly rotated black frame when $F_{a}$ changes to $F^{\prime }$, specifies the AE’s new orientation. Although during rigid rotation around the center of rotation, $C_{r}$, the points $n_{r}$ and $O_{r}$ are also translated, these displacements for the binocular system anthropomorphic dimensions listed in Figure 1 are less than 0.03 cm for typical eye rotations and are not used in geometric analysis. However, they are later used in simulations.

Referring to the figure of Section 2.2, the AE comprises the nodal point and the image plane passing through the eye’s rotation center. They represent the cornea and the crystalline lens. The image plane orientation accounts for the lens’ tilt, and the retina is replaced by its projection through the nodal point into the image plane. The projection of the fovea into the image plane determines the optical center used in this study. The eye’s misaligned optical components complicate the description of the AE’s orientation changes. In particular, the eye’s optical axis, the visual axis, the lens’s optical axis, and the fixation axis are all different (cf. Figure 1, Figure 2 and Figure 3).

Although the AE is somewhat abstract, it incorporates the eye’s anatomy and physiology (misaligned optical components that degrade visual quality but also contribute to its aplanatic design) to support the biologically mediated aspects of binocular vision that the AE aims to model [33,34,35]. In particular, in spite of the fact that the distribution of the retinal corresponding points is asymmetrical with respect to the foveae, it was demonstrated in [33] that the distribution of these corresponding points projected into the image plane of each AE is symmetric relative to the projected foveae, i.e., optical centers. Thus, unknown (asymmetric) retinal correspondence that is difficult to measure can be entirely formulated as being symmetric on the image plane of the AE in terms of its asymmetry angles. Thus, the AE model satisfies the law of parsimony: the best functional eye models include only necessary attributes for accomplishing the models’ purpose [40].

In the next section, I recall basic aspects of the AE model. It provides background to help the reader understand the rest of the paper.

## 2. Review of Asymmetric Eye Model

### 2.1. Angles of Eye’s Asymmetry

The AE was first proposed in [32] to correct and extend the classical studies on modeling empirical horopter curves as conic sections in binocular vision, first done by Ogle in 1932 [41] for symmetric fixations and later extended by Amigo in 1965 [42] to any fixations. In contrast to the modeling in [32], in their studies, the horopter curves were derived without anatomical motivation from an ad hoc equation, and their free parameters had to be determined experimentally for each subject. Also, eye movements were not included in the model.

For a comprehensive discussion of the AE model, refer to [33,35]. The typical in the human population asymmetry angles for the fovea’s horizontal and vertical displacements from the posterior pole are α=5.2° and γ=−2° and the lens’ horizontal and vertical tilts relative to the optical axis are β=3.3° and ε=−1°, as explained in Figure 1 for the right AE. The orientation of the axes determines the signs of angles. For some different values of misalignment angles, see [35].

The binocular system with the AEs has a unique finite (binocular) fixation $F_{a}$ for the given asymmetry angles α, β, γ, and ε, such that the resulting posture consists of the coplanar equatorial planes of the lenses parallel to the coplanar image planes, which align with the xz-plane in Figure 1 consisting of the head frontal plane. This is the eye’s resting posture (ERP) of the binocular system with AEs, in which the left eye is mirror-symmetric in the head’s midsagittal plane.

For the asymmetry angles listed in Figure 1, the fixation of the ERP is $F_{a}\left(0,99.56,1.72\right)$, with coordinates in centimeters matching the average abathic distance of the empirical horopter distinguished by its straight, frontal line. It numerically corresponds to the eyes’ resting vergence posture of the bifixation average distance of about 1 m, in which the eye muscles’ natural tonus resting position serves as a zero-reference level for convergence effort [43].

### 2.2. Asymmetric vs. Symmetric Eye Models

The symmetric eye (SE) shown in Figure 2a is the reduced eye with the nodal point located 0.6 cm anterior to the rotation center on the eye’s optical axis. In the SE, the asymmetry angles are zero, so that $O_{r}$, $n_{r}$, and $C_{r}$ shown in Figure 1 coincide.

The consequence of the difference between SE and AE in binocular vision is schematically shown in Figure 2c, but see [44] for a more comprehensive discussion.

As discussed in the introduction, the horopters in the binocular system with AEs resemble the empirical horopters due to the asymmetry of corresponding retinal elements, which is a direct consequence of the eye’s misaligned optical components. They are shown in Figure 2c in continuous lines. In the binocular system with SE, the horopter is the VMC, called the geometric horopter. Because of the absence of eyes’ misaligned optics in SE, there is no reason to consider the asymmetric distribution of corresponding retinal elements; the geometric horopters are circles, and, as has been commonly, but perhaps misleadingly, expressed, the empirical horopters deviate from the geometric horopters. There is also no reason to consider a special status of a unique straight-line frontal horopter at the fixation $F_{a}$, see Figure 2c.

### 2.3. Geometric Definition of Orientation Changes in AEs

The eye’s misaligned optical components complicate the description of the AE’s orientation change, which is discussed in this section. As mentioned above, the important axes of the eye differ, cf. Figure 1. Therefore, the eyeball rotation center $C_{r}$ differs from the image plane optical center $O_{r}$, where the frame that defines the AE orientation is attached, as shown in Figure 3 for the right AE. The main assumption is that when the fixation axis changes, the 3D set of the image plane, the nodal point $N_{r}$, its perpendicular projection into the image plane $n_{r}$, and the optical center $O_{r}$ rotate rigidly. I note that the line through $N_{r}$ and $n_{r}$ is the lens’s optical axis.

In Figure 3, the right AE orientation, when the eyes change the bifixation from $F_{a}$ to $F^{\prime }$, is described in terms of frame vectors at the optical center $O_{r}$.

In this figure, the black frame $\left(i_{r},j_{r},k_{r}\right)$ of the right AE is rotated into the green frame $\left(a_{r},b_{r},c_{r}\right)$ at $O_{r}$ with a corresponding rotation for the left AE when the fixation axes (the lines connecting the eyes’ rotation centers and the fixation point) throughout $F_{a}$ rotate to another fixation axis throughout $F^{\prime }$. This rotation is shown for the right eye with the angle $\Psi_{r}^{\prime }$. After rotations, the AEs’ image planes are spanned by $a_{r}$ and $c_{r}$ frame vectors for the right eye and by $a_{l}$ and $c_{l}$ vectors for the left eye. With the rigid rotations of the AEs, the frame vector normal to the image plane is parallel to the lens’s optical axis (green segments in Figure 1, Figure 2 and Figure 3).

Because the AE in Figure 3, and hence the image plane, is rigidly rotated at $C_{r}$ and the frame $\left(i_{r},j_{r},k_{r}\right)$ is attached to the image plane at $O_{r}$, the vectors are rigidly rotated and translated with the image plane. From the data shown in Figure 1, we have that the translation of $O_{r}$ is less than 0.03 cm for eye rotations less than 60° of the visual angle. However, angles of rotated vectors are unaffected by the translation. This translation is not included in Figure 3 for simplicity. Later, their translations $O_{r}^{\prime }$ and $O_{l}^{\prime }$, which are important for verifying the half-angle rule, are included in simulations discussed in Section 4.5.

## 3. Materials and Methods

The study presented here combines geometric analysis of the rotations of bifixating eyes with simulation and 3D visualization in *GeoGebra*’s dynamic geometry environment. It is supported by contemporary ophthalmology studies.

### 3.1. Ophthalmology Background: Physiological Resting Position

Twelve extraocular muscles (six attached to each eye) and a neurological control system work together to control eye position and movement. With both eyes at rest, they are directed forward in a specific position. The anatomical position, observed during deep anesthesia or death, is divergent [45]. On the other hand, the physiological position of the eyes at rest maintains a relaxed eye posture for prolonged durations without fatigue. This baseline activity level, or tonic, brings the eyes from the anatomical to the physiological resting position [46].

According to traditional theories [1,47], accommodation and vergence adjust to optical infinity in the eyes’ physiological resting posture. Helmholtz compared Listing’s Law and the primary position with parallel visual axes to the minimal-energy condition in physics. Despite this compelling argument, the modern viewpoint differs. Beginning with the suggestion by Thomas Weber in 1855 that the eye would focus at an intermediate distance when at ‘complete rest’, according to the contemporary consensus, the vergence system typically does not adjust to optical infinity (parallel gazes) at rest, but rather assumes an intermediate posture [48,49,50,51,52,53,54,55].

In these contemporary studies, the tonic vergence of the eyes at rest is measured under degraded stimulus conditions, i.e., with no stimulus for vergence or accommodation, such as in darkness or a bright, featureless field. The tonic vergence of rest measured under degraded stimulus conditions is referred to as the vergence resting position or dark vergence. This measurement varies reliably among subjects: a typical subject converges at about 1 m, while the inter-individual range spans from infinity to about 40 cm. In contrast, distance phoria (the eye’s deviation when fusion in binocular viewing is disrupted) is influenced by accommodation for the fixation target. Thus, dark vergence is a simpler index of tonic vergence.

### 3.2. Rotations with Rodrigues’ Vectors

The simulations in [35] with *GeoGebra* clearly demonstrate the power of Rodrigues’ vector (RV) framework in discussing Listing’s law in the binocular system with AEs. RV encodes both the rotation axis and rotation angle with three independent parameters—the minimal number required for rotations. It also allows for the composition of eye postures, which is crucial in formulating the binocular half-angle rule.

To introduce the RV framework, I start with the conclusion from Euler’s rotation theorem: For a rotation with one fixed point, the rotation matrix can be parametrized as R(ϕ,n) for a rotation angle ϕ around the axis with the unit vector n. Vectors are always considered in column form. This parametrization is unique if the orientation of 0<ϕ<180° is fixed. Usually, a counterclockwise (or right-hand) orientation is chosen to obtain angles with positive values.

The pair ρ=cos(ϕ/2),e=sin(ϕ/2)n is known as Euler–Rodrigues parameters and

(1) r=e/ρ=tan(ϕ/2)n

as RV, usually referred to as *rotation vector* [56]. Rodrigues proved that under composition of rotation matrices, $R\left(\phi ,n\right)=R\left(\phi^{\prime \prime },n^{\prime \prime }\right)R\left(\phi^{\prime },n^{\prime }\right)$, the corresponding Euler–Rodrigues parameters transform as follows [57],

(2) $$\begin{array}{ccc} \rho & = & \rho^{\prime }\rho^{\prime \prime }-e^{\prime }\cdot e^{\prime \prime } \end{array}$$

(3) $$\begin{array}{ccc} e & = & \rho^{\prime }e^{\prime \prime }+\rho^{\prime \prime }e^{\prime }+e^{\prime \prime }\times e^{\prime }. \end{array}$$

Setting r=e/ρ, we see after simple algebraic manipulations with Equations (2) and (3) that the composition of RVs is given by

(4) $$r=r^{\prime \prime }◦r^{\prime }=\frac{r^{\prime \prime }+r^{\prime }+r^{\prime \prime }\times r^{\prime }}{1-r^{\prime \prime }\cdot r^{\prime }}.$$

Further, the inverse of r is −r and tan(−ϕ/2)n=tan(ϕ/2)(−n) define the same rotation vector. Also, one RV corresponds to one rotation, and the RV corresponding to the unit matrix is the zero vector [56].

Note that (cos(ϕ/2),sin(ϕ/2)n) is a unit quaternion that describes the rotation by ϕ around n, and Rodrigues used in 1840 the multiplication of quaternions to obtain Equations (2) and (3). Remarkably, the quaternions were formally defined in 1843 by Hamilton, and vectors appeared late in the 19th century when Gibbs and Heaviside independently developed vector analysis. Quaternions were introduced in 1957 by Westheimer [58] to describe eye kinematics. Quaternions and Rodrigues’ (rotation) vectors have often been used to analyze the eye’s rotations with the connection to Listing’s law, cf. [5,11,12,59,60].

### 3.3. Simulation and Visualization with GeoGebra

*GeoGebra* is a free dynamic geometry software package for mathematics education research, modeling, simulation, and data visualization, particularly for solid objects and 3D geometry. It has been used in real-world modeling, such as population dynamics and traffic jam simulations; see [61], where many various modeling cases are discussed. The usefulness of *GeoGebra* lies in its excellent precision for typical 3D visualization tasks, with double-precision floating-point numbers of around 15-17 significant digits. There are many resources for *GeoGebra* usage available on the internet. A comprehensive tutorial is available at https://geogebra.github.io/docs/manual/en/, accessed on 10 March 2026.

The use of *GeoGebra* to simulate the orientation of bifixating eyes in the binocular system with AEs was pioneered by the author over the last 8 years [32,33,34,35]. The reason for using this software is its excellent dynamic 3D visualization of changes in bifixation eye orientation, which is important when the displaced fovea from the posterior pole of the eyeball and the tilted lens relative to the eye’s optical axis are taken into account. Because the eye’s imperfect optics have never been experimentally studied in binocular vision, stereopsis, and Listing’s law, the 3D visualization in *GeoGebra* provides useful information that is studied here.

*GeoGebra* simulations of the eyes’ binocular postures and related retinal coordinates in space (iso-disparity conic sections) when the eyes are rotated with an upright stationary head include geometric primitives (lines, conic sections, planes, spheres, cylinders, and the like), geometric constructs (parallel lines, perpendicular lines to planes, bisectors, and the like), and basic geometric transformations (translations, rotations, reflections, and the like). These geometric tools are used directly to design the visualization, eliminating the need for programming.

However, the primary challenge in creating a dynamic simulation for this study is connecting these geometric tools. It simulates the iso-disparity conics and the vertical horopter and computes ocular torsion. As the binocular system with AEs changes with eye rotations by moving the fixation point, *GeoGebra* visualizes the 3D transformations. In the simulations presented in this study, the number of geometric tools increases rapidly, reaching over 100 objects, whereas the objects that create connections are hidden. It hinders a straightforward understanding of the simulation construction. Moreover, a minor unintended change to any of those tools or their connections can disable the simulation. See section *Availability of data/code and materials* for more information on help with *GeoGebra* and available links to simulations performed in this study.

## 4. Results

### 4.1. Geometry of Eye’s Posture Changes and Ocular Torsion

This section discusses the precise geometry of the AE frame’s orientation change when the bifixation axes rotate from $F_{a}$ to another bifixation. The change in the eyeballs’ bifoveal fixation when eyeballs rotate about centers of rotations induces the rotations of the corresponding initial frame vectors $\left(i_{r},j_{r},k_{r}\right)$ and $\left(i_{l},j_{l},k_{l}\right)$ into the frame vectors $\left(a_{r},b_{r},c_{r}\right)$ and $\left(a_{l},b_{l},c_{l}\right)$. Because in the ERP the image planes coincide with the head frontal plane, $j_{r}=j_{l}=j$, which will be important when discussing torsion angles.

**Figure 4:**
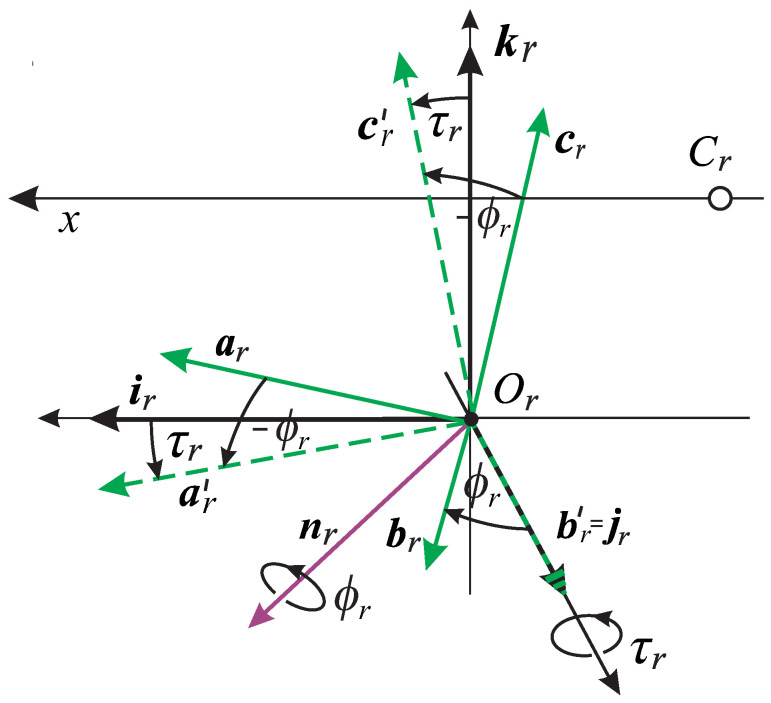
The right AE is shown in the ERP near the optical center $O_{r}$ with the transformed frames when the fixation changed from $F_{a}$. The AE’s image plane orientation is specified by the frame vectors $i_{r}$ and $k_{r}$ before rotation, which agrees with the head fronto-parallel plane (for both eyes). After rotation, the image plane is spanned by $a_{r}$ and $c_{r}$. The RV $r_{r}=\tan\left(\phi_{r}/2\right)n_{r}$ (vector shown in purple) rotates $j_{r}$ onto $b_{r}$ such that the axis of rotation $n_{r}$ is perpendicular to both vectors. When the RV $-r_{r}=\tan\left(-\phi_{r}/2\right)n_{r}$ is applied to the whole frame $\left(a_{r},b_{r},c_{r}\right)$, the result is the frame $\left(a_{r}^{\prime },j,c_{r}^{\prime }\right)$. Since the vectors $a_{r}^{\prime }$ and $c_{r}^{\prime })$ are perpendicular to $j_{r}$, the angle of ocular torsion satisfies $\tau_{r}=∠\left(i_{r},a_{r}^{\prime }\right)=∠\left(k_{r},c_{r}^{\prime }\right)$. Similar relations hold for the left eye torsion under the condition that $j_{r}=j_{l}$ in ERP. This follows from the condition that, in ERP, the image planes of both eyes coincide with the head’s fronto-parallel plane such that the orthogonal vectors $j_{r}$ and $j_{l}$ also coincide.

The main result presented in Figure 4 and confirmed by simulations shown in the next section, is that the change in positions of the rotated AE is uniquely split into two parts: the torsion-free and the ocular torsion rotations. By referring to Figure 4, I first define for the right AE the rotation of $j_{r}$ to $b_{r}$ in the plane spanned by these two vectors by the angle $\phi_{r}$. Thus, the axis of this rotation, $n_{r}$, is perpendicular to both vectors, such that the corresponding RV is $r_{r}=\tan\left(\phi_{r}/2\right)n_{r}$. By this definition, the $n_{r}$ trace out an arc of a great circle on a unit sphere centered at $O_{r}$ for the right eye when the eye rotates. This path gives a geodesic on the manifold of the rotation group SO(3) [62]. I will demonstrate in Section 4.5 that this rotation precisely describes the direction change of the visual axis of the right eye. The same holds for the left eye, such that the bifixation is preserved.

The inverse rotations by RVs $-r_{r}=\tan\left(-\phi_{r}/2\right)n_{r}$ and $-r_{l}=\tan\left(-\phi_{l}/2\right)n_{l}$ when applied to the whole frames $\left(a_{r},b_{r},c_{r}\right)$ and $\left(a_{l},b_{l},c_{l}\right)$ produce the frames $\left(a_{r}^{\prime },j,c_{r}^{\prime }\right)$ and $\left(a_{l}^{\prime },j,c_{l}^{\prime }\right)$, respectively, (because $r_{r}$ rotates j to $b_{r}$ and $r_{l}$ rotates j to $b_{l}$). It shows that the ‘primed’ frames rotate around the vector j, which remains unchanged. These rotations define the torsional parameters of AEs given by angles $\tau_{r}=∠\left(k_{r},c_{r}^{\prime }\right)$ for the right eye and $\tau_{l}=∠\left(k_{l},c_{l}^{\prime }\right)$ for the left eye. Because vector j for each eye is parallel to the lens’ optical axis at the distance of 0.02 cm (cf. Figure 1), the rotations very closely represent the torsional components of the eye movements around these optical axes.

As mentioned above, the torsion-free RVs $r_{r}=\tan\left(\phi_{r}/2\right)n_{r}$ for the right AE lie within the ERP plane, which serves as the Listing plane. The torsional RV $q_{r}=\tan\left(\tau_{r}/2\right)j$, on the other hand, rotates the right AEs about the vector perpendicular to the ERP plane, which coincides with the head’s frontal plane and is parallel to the lens’s optical axis of each eye.

Ocular torsion, which is important for clinical diagnosis [63], has been traditionally measured using the 3D search-coil method developed in [64]. More recently, it is often measured using video-oculography as a non-invasive, high-resolution technique. A robust method for measuring ocular torsion using this technique was proposed in [65,66], for example, which tracks the angular shift of the iris pattern about the pupil center. Thus, the geometric parametrization of ocular torsion in this study is more consistent with that used in clinical diagnosis.

The decomposition of the AEs rotations into the torsion-free and torsional parts indicates that the 3D eye rotations of binocularly constrained fixations are geometrically coupled, meaning that non-zero ocular torsion is almost always present for all eye rotations. Therefore, this decomposition will be crucial for demonstrating Listing’s law and the half-angle rule, and for formulating their configuration space and discussing noncommutativity in Section 4.6.

### 4.2. Simulations of Torsion-Free and Torsional RVs

**Figure 5:**
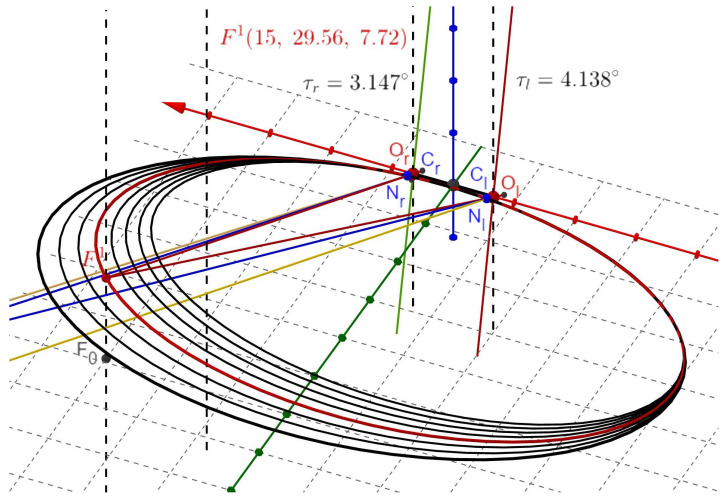
Simulated iso-disparity curves when $F_{a}\left(0,99.56,1.72\right)\to F^{1}\left(15,29.56,7.72\right)$. The iso-disparity curves are ellipses with the horopter in red passing through the fixation point $F^{1}$. The red horizontal line is the *x*-axis, with the green and blue lines denoting the *y*- and *z*-axes. All dashed lines show the vertical direction, and the green and brown lines are perpendicular to the plane containing the iso-disparity ellipses. The right AE ocular torsion is $\tau_{r}=3.147{}^{\circ}$ and the left AE is $\tau_{l}=4.138{}^{\circ}$. The details of the construction of the longitudinal iso-disparity curves that include ellipses, straight fronto-paralllel lines and hyperbolas, all resembling empirical iso-disparity curves, are given in [35].

**Figure 6:**
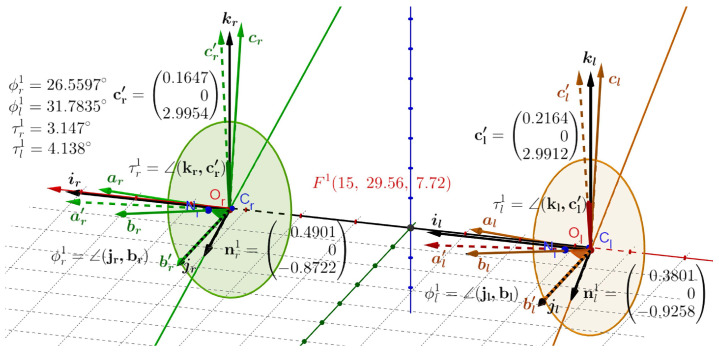
Simulated RVs $r_{r}^{1}=\tan\left(\phi_{r}^{1}/2\right)n_{r}^{1}$, $r_{l}^{1}=\tan\left(\phi_{l}^{1}/2\right)n_{l}^{1}$ and $q_{r}^{1}=\tan\left(\tau_{r}^{1}/2\right)j_{r}$, $q_{l}^{1}=\tan\left(\tau_{l}^{1}/2\right)j_{l}$ when $F_{a}\to F^{1}$. The black frames in the ERP $\left(i_{r},j_{r},k_{r}\right)$ and $\left(i_{l},j_{l},k_{l}\right)$ for the right and left AEs are attached at the image planes’ optical centers $O_{r}$ and $O_{l}$. Their orientations agree with the upright head’s coordinate system. The solid-green frame $\left(a_{r},b_{r},c_{r}\right)$ for the right eye and the solid-brown frame $\left(a_{l},b_{l},c_{l}\right)$ for the left eye are moving from the initial position of the black frames when fixation $F_{a}$ changes to fixation $F^{1}$. The dashed-colored frames are obtained by rotating the solid-colored frames by moving the b vectors onto the j vectors about the angles $-\phi_{r}^{1}$ and $-\phi_{l}^{1}$ around the rotation axes $n_{r}^{1}$ and $n_{l}^{1}$ for the corresponding eyes. They lie on the co-planar image planes of the ERP, which coincide with the head’s frontal plane. The simulated ocular torsion is $\tau_{r}^{1}=∠{(k}_{r},{c^{\prime }}_{r})=∠{(i}_{r},{a^{\prime }}_{r})$ for the right eye and $\tau_{l}^{1}=∠{(k}_{l},{c^{\prime }}_{l})=∠{(i}_{l},{a^{\prime }}_{l})$ for the left eye as explained in Figure 4. The circles represent the cross-sections of the AEs by the image planes. The frame vectors are 3 cm long for clarity in the picture.

The RV for each eye is computed, simulated, and visualized in *GeoGebra* for rotations about the ERP, and the binocular substitution for the original single-eye Listing’s plane. Before reading this section, it is advisable to review the simulations presented in [35] to see how the simulations given below are related to other basic simulation cases. The simulation in Figure 5 shows the iso-disparity curves for the change of the fixation from $F_{a}$ to $F^{1}\left(15,29.56,7.72\right)$ with calculated torsion for AEs. The zoom from Figure 5 to Figure 6 demonstrates the power of *GeoGebra*, which can be used to set a scale showing 0.5 m, then zoom to a scale showing 1 mm.

The first simulation is shown in Figure 6 for the change of bifixation at $F_{a}\left(0,99.56,1.72\right)$ of the ERP to tertiary posture with the bifixation at $F^{1}\left(15,29.56,7.72\right)$. In this figure, when $F_{a}$ changes to $F^{1}$, the solid green frame for the right AE, $\left(a_{r},b_{r},c_{r}\right)$, and the brown frame for the left AE, $\left(a_{l},b_{l},c_{l}\right)$, are moving with the image planes from their position that initially in ERP agrees with the black frames, $\left(i_{r},j_{r},k_{r}\right)$ and $\left(i_{l},j_{l},k_{l}\right)$. From the definition of the ERP, the corresponding pairs of vectors of the initial frames are the same. As mentioned before, although I use subscripts to indicate the right or left eye, I sometimes explicitly write $j_{r}=j_{l}=j$ when discussing the torsional RVs. In the simulations, the length of the frame vectors is set to 3 cm to improve visualization.

When the fixation axes that connect the eyes’ rotation centers $C_{r}$ and $C_{l}$ with $F_{a}$ change direction (cf. Figure 3), the initial black frames attached to the optical centers $O_{r}$ and $O_{l}$ for the left and right AEs rotate with the corresponding image planes. The RV rotating $j_{r}$ onto $b_{r}$ and $j_{l}$ onto $b_{l}$ by computed angles $\phi_{r}^{1}=26.56{}^{\circ}$ and $\phi_{l}^{1}=31.78{}^{\circ}$ in the plane spanned by both vectors for each AE precisely describes the change in orientation of their visual axes. These facts are demonstrated in Figure 6. These RVs are

(5) $$r_{r}^{1}=\tan\left(\frac{\phi_{r}^{1}}{2}\right)n_{r}^{1}\;\;\mathrm{and}\;\;r_{l}^{1}=\tan\left(\frac{\phi_{l}^{1}}{2}\right)n_{l}^{1}$$

for the right and left eyes. The vectors $n_{r}^{1}$ and $n_{l}^{1}$ are perpendicular to both $j_{r}$, $b_{r}$ and $j_{l}$, $b_{l}$. Therefore, each lies in its respective AE’s image plane, which aligns with the head’s frontal plane and serves as the binocular Listing plane. As mentioned before, each of the RVs in Equation (5) represents the shortest path on the rotation group SO(3), i.e., the geodesic [62].

To calculate each AE ocular torsion, the green and brown frames are rotated back, reversing the rotations that overlaid js vectors with bs vectors. These rotation angles around $n_{r}^{1}$ and $n_{l}^{1}$ are $-\phi_{r}^{1}=∠\left(b_{r},j_{r}\right)=-26.560{}^{\circ}$ and $-\phi_{l}^{1}=∠\left(b_{l},j_{l}\right)=-31.784{}^{\circ}$. When these rotations are applied to the whole colored frames, they result in two ‘primed’ frames in dashed lines, the green frame $\left(a_{r}^{\prime },j,c_{r}^{\prime }\right)$, and the brown frame $\left(a_{l}^{\prime },j,c_{l}^{\prime }\right)$. The last rotations lead to the definition of ocular torsion and its value for each eye, $\tau_{r}^{1}=∠{(k}_{r},{c^{\prime }}_{r})=∠{(i}_{r},{a^{\prime }}_{r})=3.15{}^{\circ}$ and $\tau_{l}^{1}=∠{(k}_{l},{c^{\prime }}_{l})=∠{(i}_{l},{a^{\prime }}_{l})=4.14{}^{\circ}$. Thus, the torsional disparity $\delta_{t}^{1}=\tau_{r}^{1}-\tau_{l}^{1}\approx -0.99{}^{\circ}$.

**Figure 7:**
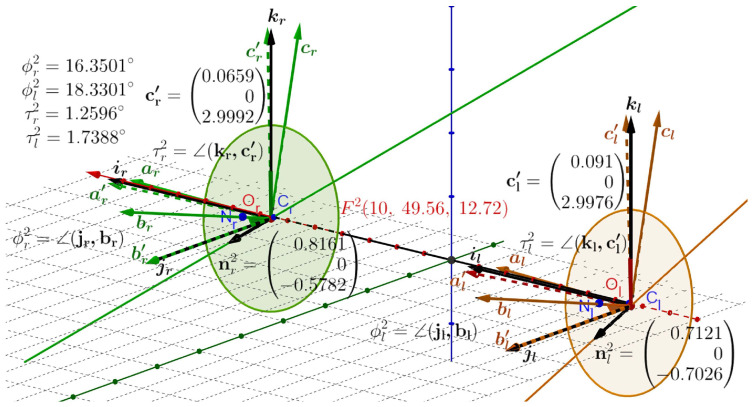
RVs $r_{r}^{2}=\tan\left(\phi_{r}^{2}/2\right)n_{r}^{2}$, $r_{l}^{2}=\tan\left(\phi_{l}^{2}/2\right)n_{l}^{2}$ and $q_{r}^{2}=\tan\left(\tau_{r}^{2}/2\right)j_{r}$, $q_{l}^{2}=\tan\left(\tau_{l}^{2}/2\right)j_{l}$ when $F_{a}\to F^{2}$. A similar discussion as in Figure 6 applies here. For the fixation $F^{2}(10,49.56,12.72$, the frames’ angles of rotations around the axes $n_{r}^{2}$ and $n_{l}^{2}$ and ocular torsion angles of each eye are given in the figure.

Similarly to above, from the data in Figure 7, when $F_{a}\left(0,99.56,1.72\right)$ changes to $F^{2}\left(10,49.56,12.72\right)$, we obtain the RVs describing the direction changes of the visual axes,

(6) $$r_{r}^{2}=\tan\left(\frac{\phi_{r}^{2}}{2}\right)n_{r}^{2}\;\;\mathrm{and}\;\;r_{l}^{2}=\tan\left(\frac{\phi_{l}^{2}}{2}\right)n_{l}^{2},$$

and the simulated ocular torsion of the right eye $\tau_{r}^{2}=∠{(k}_{r},{c^{\prime }}_{r})=∠{(i}_{r},{a^{\prime }}_{r})=1.26{}^{\circ}$ and the left eye is $\tau_{l}^{2}=∠{(k}_{l},{c^{\prime }}_{l})=∠{(i}_{l},{a^{\prime }}_{l})=1.74{}^{\circ}$. Here, torsional disparity is $\delta_{t}^{2}=\tau_{r}^{2}-\tau_{l}^{2}=-0.52{}^{\circ}$.

### 4.3. Discussion of Simulations

Referring to Figure 3, when the right eye fixation axis given by vector $V_{ar}=\vec{C_{r}F_{a}}$ changes to the fixation axis given by vector $V_{r}^{1}=\vec{C_{r}F^{1}}$, the RV can be computed as follows. The angle between the fixation axes is

(7) $$\Psi_{r}^{1}=\cos^{-1}\;\left(\frac{V_{ar}\cdot V_{r}^{1}}{∥V_{ar}∥∥V_{r}^{1}∥}\right)$$

such that Rodrigues’ vector is $Q_{r}^{1}=\tan\left(\Psi_{r}^{1}/2\right)N_{r}^{1}$ where

(8) $$N_{r}^{1}=\frac{V_{ar}\times V_{r}^{1}}{∥V_{ar}\times V_{r}^{1}∥}.$$

is the unit vector of the rotation axis. RV for the left eye, $Q_{l}^{1}$, is similarly defined.

Taking the values $F_{a}\left(0,99.56,1.72\right)$, $C_{r}\left(3.25,0,0\right)$ and $F^{1}\left(a,b,c\right)$ in centimeters, we obtain

(9) $$\frac{V_{ar}\cdot V_{r}^{1}}{∥V_{ar}∥∥V_{r}^{1}∥}=\frac{-3.25a+99.56b+1.72c+10.5625}{99.628\sqrt{{\left(a-3.25\right)}^{2}+b^{2}+c^{2}}}$$

and

(10) $$V_{a}\times V_{r}^{1}={\left(99.56c-1.72b,3.25c+1.72a-5.59,-3.25b-99.56a+323.57\right)}^{t},$$

which are used to get Equations (7) and (8). The superscript "*t*" means the transpose, which produces column vectors.

For the two simulations carried out in Section 4.2, RVs are computed from simple algebraic and trigonometric Equations (7)–(10) for the indicated fixation $F^{1}$ and $F^{2}$ as follows:

1. For $F^{1}\left(15,29.56,7.72\right)$, $Q_{r}^{1}=\tan\left(26.49{}^{\circ}/2\right){\left(0.493,0.031,-0.869\right)}^{t}$
2. For $F^{2}\left(10,49.56,12.72\right)$, $Q_{r}^{2}=\tan\left(16.26{}^{\circ}/2\right){\left(0.817,0.037,-0576\right)}^{t}$.

The corresponding simulated RVs were $r_{r}^{1}=\tan\left(26.56{}^{\circ}/2\right){\left(0.4901,0,-0.8727\right)}^{t}$ and $r_{r}^{2}=\tan\left(16.35{}^{\circ}/2\right){\left(0.8161,0,-0.5782\right)}^{t}$. The difference of $V_{a}$ from the perpendicularity by about 2° explains the most significant differences (although in tenths of millimeters) in the second vectors’ components between Qs vectors and rs vectors.

### 4.4. RVs Between Eyes’ Tertiary Positions

The eyes’ rotations are obtained between the tertiary positions from $F^{1}$ to $F^{2}$ in the framework of RVs. This leads to the binocular half-angle rule, which is later confirmed by *GeoGebra* simulations. Listing’s law and the half-angle rule are only approximate, whether investigated experimentally (as has been done so far) or tested in modeling that includes the eye’s misaligned optical components (as done here). The key to demonstrating the half-angle rule here is the decomposition of the eye rotations as described by the torsion-free RVs and torsional RVs. The eye’s change in tertiary position is first discussed for the geodesic part of torsion-free rotation of the visual axis, followed by the torsional rotation about the lens’s optical axis.

From the definitions of RV in Equation (1) and their composition in Equation (4), the sequence of the changes in the fixations $F_{a}\to F^{1}\to F^{2}$ results in the composition of RVs for the respective eyes:

$$r_{r}^{12}◦r_{r}^{1}=r_{r}^{2}\;\;\;\mathrm{and}\;\;\;r_{l}^{12}◦r_{l}^{1}=r_{l}^{2}.$$

Here, RVs $r_{r}^{12}$ and $r_{l}^{12}$ correspond to the right and left eyes’ rotations when bifoveal fixation $F^{1}$ changes to $F^{2}$ in the stationary upright head. Also, RVs $r_{r}^{i}$ and $r_{l}^{i}$ correspond to the change $F_{a}\to F^{i}$, where $F_{a}$ is fixation of the ERP and i=1,2.

Using that $r^{-1}=-r$, the above equations written as follows:

(11) $$r_{r}^{12}=r_{r}^{2}◦-r_{r}^{1}\;\;\;\mathrm{and}\;\;\;r_{l}^{12}=r_{l}^{2}◦-r_{l}^{1},$$

are compositions of RVs for the right and the left eye corresponding to the composition of rotations $R_{2}\left(\phi_{r}^{2},n_{r}^{2}\right)R_{1}\left(\phi_{r}^{1},-n_{r}^{1}\right)$ and $R_{2}\left(\phi_{l}^{2},n_{l}^{2}\right)R_{1}\left(\phi_{l}^{1},-n_{l}^{1}\right)$ when bifixation changes from $F^{1}$ and $F^{2}$.

Using Equations (4) and (11) to obtain RVs $r_{r}^{12}$ and $r_{l}^{12}$, then introducing simulations of Equations (5) and (6) and, finally, calculating the cross and scalar products of RVs $r_{r}^{12}$ and $r_{l}^{12}$ are the following:

(12) $$\begin{array}{ccc} r_{r}^{12} & = & \frac{r_{r}^{2}-r_{r}^{1}-r_{r}^{2}\times r_{r}^{1}}{1+r_{r}^{2}\cdot r_{r}^{1}}={\left(0.00152,-0.01410,0.11920\right)}^{t} \end{array}$$

(13) $$\begin{array}{ccc} r_{l}^{12} & = & \frac{r_{l}^{2}-r_{l}^{1}-r_{l}^{2}\times r_{l}^{1}}{1+r_{l}^{2}\cdot r_{l}^{1}}={\left(0.00640,-0.01728,0.14412\right)}^{t}, \end{array}$$

and we obtain

$$\begin{array}{ccc} \tan\frac{\phi_{r}^{12}}{2} & = & ∥r_{r}^{12}∥=0.12004\\ \tan\frac{\phi_{l}^{12}}{2} & = & ∥r_{l}^{12}∥=0.14529, \end{array}$$

which give the corresponding angle values $\phi_{r}^{12}=13.690{}^{\circ}$ and $\phi_{l}^{12}=16.534{}^{\circ}$ for the right and left eyes, respectively.

Further, following similar lines as before to obtain Equations (12) and (13),

(14) $$\begin{array}{ccc} r_{r}^{21} & = & \frac{r_{r}^{1}-r_{r}^{2}-r_{r}^{1}\times r_{r}^{2}}{1+r_{r}^{2}\cdot r_{r}^{1}}=-r_{r}^{12} \end{array}$$

(15) $$\begin{array}{ccc} r_{l}^{21} & = & \frac{r_{l}^{1}-r_{l}^{2}-r_{l}^{1}\times r_{l}^{2}}{1+r_{l}^{2}\cdot r_{l}^{1}}=-r_{l}^{12}. \end{array}$$

Similarly, I describe the changes in the torsion parameters encoded by RV $q_{r}=\tan\left(\tau_{r}/2\right)j$ for the right eye and $q_{l}=\tan\left(\tau_{r}/2\right)j$ for the left eye. I denote the corresponding changes between the tertiary positions for the right eye by RV $q_{r}^{12}$, so that $q_{r}^{12}◦q_{r}^{1}=q_{r}^{2}$. Using that the RV inverse satisfies $r^{-1}=-r$, we have $q_{r}^{12}=q_{r}^{2}◦-q_{r}^{1}$. Using Equation (4) and the identity j×j=0, we obtain

(16) $$\begin{array}{ccc} q_{r}^{12} & = & \frac{q_{r}^{2}-q_{r}^{1}-q_{r}^{2}\times q_{r}^{1}}{1+q_{r}^{2}\cdot q_{r}^{1}} \end{array}$$

(17) $$\begin{array}{ccc} & = & \frac{(\tan\left(\tau_{r}^{2}/2\right)-\tan\left(\tau_{r}^{1}/2\right))j}{1+\tan\left(\tau_{r}^{2}/2\right)\tan\left(\tau_{r}^{1}/2\right)} \end{array}$$

(18) $$\begin{array}{ccc} & = & \tan(\tau_{r}^{2}-\tau_{r}^{1})j. \end{array}$$

The last equality uses a standard trigonometric identity. Along similar lines, we obtain

(19) $$q_{l}^{12}=\tan\left(\tau_{l}^{2}-\tau_{l}^{1}\right)j$$

for the left eye. Formulas (18) and (19) allow the use of the notation $\tau_{r}^{12}=\tau_{r}^{2}-\tau_{r}^{1}$ and $\tau_{l}^{12}=\tau_{l}^{2}-\tau_{l}^{1}$ for the corresponding eyes. Thus, the disparity of torsion pa, using ERP, satisfies the relation

(20) $$\begin{array}{ccc} \delta_{t}^{12} & = & \left(\tau_{r}^{2}-\tau_{r}^{1}\right)-\left(\tau_{l}^{2}-\tau_{l}^{1}\right)=\tau_{r}^{12}-\tau_{l}^{12} \end{array}$$

(21) $$\begin{array}{ccc} & = & \left(\tau_{r}^{2}-\tau_{l}^{2}\right)-\left(\tau_{r}^{1}-\tau_{l}^{1}\right)=\delta_{t}^{2}-\delta_{t}^{1}. \end{array}$$

It is now straightforward to obtain

(22) $$\begin{array}{ccc} q_{r}^{21} & = & \tan\left(\tau_{r}^{1}-\tau_{r}^{2}\right)j=-q_{r}^{12} \end{array}$$

(23) $$\begin{array}{ccc} q_{l}^{21} & = & \tan\left(\tau_{l}^{1}-\tau_{l}^{2}\right)j=-q_{l}^{12}, \end{array}$$

such that $\tau_{t}^{21}=-\tau_{t}^{12}$ and $\delta_{t}^{21}=-\delta_{t}^{12}$.

### 4.5. Simulation of the Half-Angle Rule

**Figure 8:**
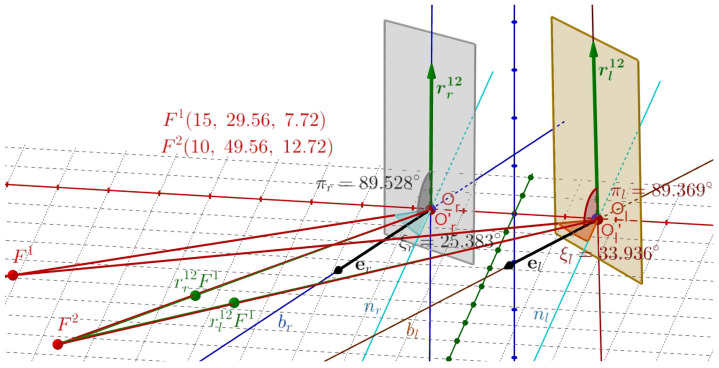
The half-angle rule for $F^{1}\to F^{2}$. The green dots $r_{r}^{12}F^{1}$ and $r_{l}^{21}F^{1}$ are the rotations of $F^{1}$ by the indicated RVs. The green lines through the green dots and $O_{r}^{\prime }$ and $O_{l}^{\prime }$ overlying the visual axes of $F^{2}$ are obtained by these RVs; they generate the eyes’ rotations from $F^{1}$ to $F^{2}$. The vectors $e_{r}$ and $e_{l}$ are bisectors between lines $n_{r}$ and $n_{l}$ perpendicular to the ERP and the visual axes of $F^{1}$ for the left and right AE. The displacement planes are perpendicular to the bisectors and are shown as rectangular areas. Consistent with the half-angle rule, the RVs are well approximated to lie within the displacement planes. The approximations result from the eye’s misalignment of the optical components.

The binocular half-angle rule between tertiary positions in the binocular system with AEs is formulated above in terms of RVs for rotations and using ERP for initial and terminal fixations. It is demonstrated in this section using *GeoGebra* simulations shown in Figure 6 and Figure 7. Let $F_{a}\to F^{1}\to F^{2}$. In Figure 8, we see the visual axes through $F^{1}$ and $O_{r}$, denoted below by $l_{r}$, and through $F^{1}$ and $O_{l}$ by $l_{l}$.

Furthermore, we see the normal lines to ERP planes, $n_{r}$ and $n_{l}$, passing through $O_{r}$ and $O_{l}$, respectively. In *GeoGebra*, we construct the bisector $b_{r}$ between lines $l_{r}$ and $n_{r}$ and the bisector $b_{l}$ between lines $l_{l}$ and $n_{l}$. The displacement planes shown as colored rectangles for the half-angle rule when $F^{1}\to F^{2}$ are planes that are perpendicular to the bisectors, one passing through $O_{r}$ and the other by $O_{l}$. The calculated RVs $r_{r}^{12}$ and $r_{l}^{12}$ lie within the respective displacement planes.

This construction demonstrates the half-angle rule for $F^{1}\to F^{2}$ as follows. The green dots $r_{r}^{12}F^{1}$ and $r_{l}^{21}F^{1}$ in Figure 8 are the rotations of $F^{1}$ by the indicated RVs. The green lines through the green dots and $O_{r}^{\prime }$ and $O_{l}^{\prime }$ overlying the visual axes of $F^{2}$, shown in *GeoGebra* simulation, represent the eyes’ rotations from $F^{1}$ to $F^{2}$. The points $O_{r}^{\prime }$ and $O_{l}^{\prime }$ are translated points $O_{r}$ and $O_{l}$ by less than 0.03 cm when the eyes are rotated at $C_{r}$ and $C_{l}$, as discussed before.

**Figure 9:**
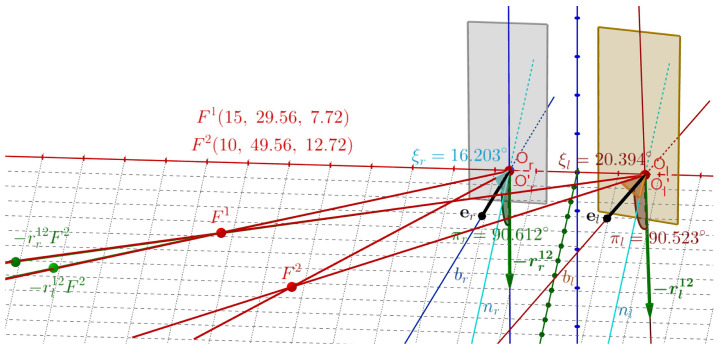
The half-angle rule for $F^{2}\to F^{1}$. Similarly to the discussion in the caption of Figure 8, green dots $r_{r}^{21}F^{2}=-r_{r}^{12}F^{2}$ and $r_{l}^{21}F^{2}=-r_{l}^{12}F^{2}$ are the rotations of $F^{2}$ by the indicated RVs. The green lines through the green dots, $O_{r}$, and $O_{l}$, overlay the visual axes of $F^{1}$ for the right and left eyes. Notation is similar as in Figure 8.

In Figure 9, the demonstration of the half-angle rule for $F_{a}\to F^{2}\to F^{1}$ is given along similar lines as above.

The simulations demonstrate that the computed RVs $r_{r}^{21}=-r_{r}^{12}$ and $r_{l}^{21}=-r_{l}^{12}$ lie in the corresponding displacement planes determined by the bisectors in Figure 9. It also verifies that these RVs generate the fixation $F^{1}$ by rotating $F^{2}$; the lines through the green dots $-r_{r}^{12}F^{2}$ and $-r_{l}^{12}F^{2}$ and the points $O_{r}^{\prime }$ and $O_{l}^{\prime }$ perfectly overlying the red visual axes of $F_{1}$. I conclude that the above constructions in Figure 8 and Figure 9 demonstrate the *binocular half-angle rule*.

The displacement planes (shown in the figures as rectangular areas) are defined for the respective eyes by the vectors $e_{r}$ and $e_{l}$ along the bisectors. The angles between $r_{r}^{12}$ and $e_{r}$ have the value of $\pi_{r}=89.53{}^{\circ}$ and between $r_{l}^{12}$ and $e_{l}$ the value of $\pi_{l}=89.37{}^{\circ}$ in the first simulation and the angles between $-r_{r}^{12}$ and $e_{r}$ have the value of $\pi_{r}=90.61{}^{\circ}$ and between $-r_{l}^{12}$ and $e_{l}$ the value of $\pi_{l}=90.52{}^{\circ}$ in the second simulation. It shows approximations of the half-angle rule for each eye due to misalignment of the eye’s optical components.

Nevertheless, the precise noncommutative behavior underlying the half-angle rule was never formulated in a definitive manner; it was instead loosely attributed to the noncommutativity of 3D rotations. Based on the torsion-free and torsional decompositions of eye-position changes, the composition of RVs, and the binocular half-angle rule demonstrated in simulations, the configuration space of the sequences of bifixation changes is next discussed.

### 4.6. The Binocular Configuration Space

The eyes’ configuration specifies their stationary bifoveal posture in the binocular system of a stationary upright head. The element of this configuration space (CS) for each eye comprises pairs of torsion-free rotations of the change in the visual axis direction and the change in torsion angles, both rotations represented by RVs. I recall that RV has three independent parameters, the minimal number needed to uniquely specify the rotation, which is particularly well-suited for formulating the CS.

Thus, the CS consists of pairs of RVs $\sigma_{r}^{m}=\left(r_{r}^{m},q_{r}^{m}\right)$ for the right AE and $\sigma_{l}^{m}=\left(r_{l}^{m},q_{l}^{m}\right)$ for the left AE, which represent rotations from fixation $F_{a}$ of the ERP to $F^{m}$. For the change of fixation $F^{m}\to F^{n}$ between tertiary postures, $\sigma_{r}^{mn}\equiv \left(r_{r}^{mn},q_{r}^{mn}\right)$ and $\sigma_{l}^{mn}\equiv \left(r_{l}^{mn},q_{l}^{mn}\right)$ for the corresponding eyes. I recall that for $F^{n}\to F^{m}$, $\sigma_{r}^{nm}=-\sigma_{r}^{mn}$ and similarly for the left eye.

Further, I define $\sigma^{mn}=\left(\sigma_{r}^{mn},\sigma_{l}^{mn}\right)$ and the operation

(24) $$\begin{array}{c} \sigma^{kl}◦\sigma^{lm}=\left(\sigma_{r}^{kl},\sigma_{l}^{kl}\right)◦\left(\sigma_{r}^{lm},\sigma_{l}^{lm}\right)=\left(\sigma_{r}^{kl}◦\sigma_{r}^{lm},\sigma_{l}^{kl}◦\sigma_{l}^{lm}\right). \end{array}$$

as follows:

(25) $$\begin{array}{ccc} \sigma_{r}^{kl}◦\sigma_{r}^{lm} & = & \left(r_{r}^{kl},q_{r}^{kl}\right)◦\left(r_{r}^{lm},q_{r}^{lm}\right)\\ & = & \left(r_{r}^{kl}◦r_{r}^{lm},q_{r}^{kl}◦q_{r}^{lm}\right) \end{array}$$

and similarly for $\sigma_{l}^{kl}◦\sigma_{l}^{lm}$.

Now, the binocularly constrained eyes’ changes involving fixations $F_{a}$, $F^{k}$, $F^{l}$, and $F^{m}$ are the following:

(26) $$\begin{array}{ccccccc} \; & F^{k} & \;\overset{\sigma^{kl}}{\underset{{-\sigma }^{kl}}{↔}}\; & F^{l} & \;\overset{\sigma^{lm}}{\underset{\sigma^{lm}}{↔}}\; & F^{m} & \\ \cdots & \;\sigma^{k}↕-\sigma^{k} & \; & \;\sigma^{l}↕-\sigma^{l} & \; & \;\sigma^{m}↕-\sigma^{m} & \cdots \\ \; & F_{a} & \overset{=}{↔} & F_{a} & \overset{=}{↔} & F_{a} \end{array}$$

where the minus sign indicates a down arrow or a left arrow.

To simplify the discussion, I introduce the short notation $r^{mn}=\left(r_{r}^{mn},r_{l}^{mn}\right)$ with the operation

(27) $$\left(r_{r}^{kl},r_{l}^{kl}\right)◦\left(r_{r}^{lm},r_{l}^{lm}\right)=\left(r_{r}^{kl}◦r_{r}^{lm},r_{l}^{kl},r_{l}^{lm}\right)$$

and discuss only its compositions. The composition of q-terms is simple, such that this simplified presentation can be easily extended to $\sigma^{mn}$. The composition is associative, for example,

(28) $$\left(r^{kl}◦r^{lm}\right)◦r^{mn}=r^{kl}◦\left(r^{lm}◦r^{mn}\right)=r^{kl}◦r^{lm}◦r^{mn}.$$

It is noncommutative, but with a simple noncommutativity rule for Listing’s law. For example,

(29) $$r^{kl}=r^{l}◦-r^{k}=-\left(r^{k}◦-r^{l}\right)=-r^{lk}.$$

Note that $r^{kl}$ transforms the fixation from $F^{k}$ to $F^{l}$ and $-r^{kl}$ transforms from $F^{l}$ to $F^{k}$; cf. Figure 8 and Figure 9.

Finally, let’s examine the path: $F^{m}\to F_{a}\to F^{l}\to F^{k}$. The composition of the corresponding RVs is

(30) $$r^{lk}◦r^{l}◦-r^{m}=r^{k}◦-r^{l}◦r^{l}◦-r^{m}=r^{k}◦-r^{m}=r^{mk}.$$

Recall that the composition order of RVs is the same as the composition order of the corresponding rotation matrices, i.e., from right to left.

## 5. Discussion

Listing’s law is a preferred 3D rule for eye orientation and holds approximately during fixation, saccades, smooth pursuit, and vergence, but not during sleep and vestibulo-ocular reflex. It suggests that it is actively implemented by a neural mechanism. It is executed by the six oculomotor muscles in each eye and may also be supported by orbital constraints such as pulleys. Listing’s law and the related half-angle rule may simplify visual processing and optimize oculomotor efficiency. Importantly, it also has clinical implications for the optimal management of strabismus disorders.

Listing’s law is simple to state geometrically but much harder to interpret biologically, especially as revealed by the clinical investigations discussed below. Not surprisingly, many deficiencies of Listing’s law have been discussed in the literature, as reviewed in the Introduction. In the study presented here, Listing’s law formulation accounts for the misalignment of the optical components of the healthy human eye. It makes the analysis more complicated, mainly because all standard eye axes differ. However, the outcome is the ab initio formulation of Listing’s law and the related half-angle rule, which is confirmed by simulations of kinematic changes of binocularly constrained eyes. In this formulation, Listing’s plane of the eye’s primary position is replaced by the ERP, the binocular eyes’ posture corresponding to the eye muscles’ natural tonus resting position, which serves as a zero-reference level for convergence effort. Further, it corresponds to the unique abathic distance fixation for empirical horopters distinguished by their straight-line shape.

### 5.1. ERP Substitute for the Primary Position of Listing’s Law

The estimation of the range of ERP’s fixation point $F_{a}$ using Equation (3) in [33] for horizontal misalignment (no vertical misalignment) was as follows: for the average values of the interocular distance 2a=6.5 cm and the angles α=5.2° and −0.4°≤β≤4.7°, we obtain the distance to the ERP between 34 and 380 in centimeters with the average of about 1 m for the typical value β=3.3°. More precisely, the fixation point of ERP is $F_{a}\left(0,99.61,0\right)$, without vertical misalignment included. It was also noted that this distance to $F_{a}$ approaches infinity (as the value of β approaches that of α), as reported in some experimental measurements. I conclude that the binocular ERP not only provides the necessary neurophysiological meaning for the elusive significance of primary position and Listing plane in oculomotor control [16] but also supersedes the original monocular Listing’s law with the ab initio formulated binocular law.

In a 3D setting of the binocular system with AEs, the image planes for the ERP binocular posture are coplanar and parallel to the coplanar lenses’ equatorial planes and constitute the frontal plane of the stationary upright head. When the eyes rotate from the ERP, the image planes move with the AE remaining parallel to each lens’s equatorial plane. Given the above discussion, I replace the Listing plane with the head frontal pane. The fixation axis of each AE with the asymmetry angles α=5.2°, β=3.3°, γ=−2°, and ε=−1° in this posture passes through the point $F_{a}\left(0,99.56,1.72\right)$, expressed in centimeters, and is not perpendicular to the frontal plane. I note that the added vertical misalignment angles slightly changed the position of $F_{a}$, but not its distance.

The precise results on the lack of perpendicularity are obtained by calculations using Equation (3) in [33] and the dot product of the vectors $j_{r}$ and $\vec{C_{r}F_{a}}$ to compute the angle between them (see Figure 3 and Table 2 in [35]). Thus, in the binocular system with AEs, the Listing plane is substituted by the ERP, and the eye’s primary direction deviates from perpendicularity by about 2.12° for the average asymmetry angles given above, and for the range of 0°≤β≤4.5°, it deviates from perpendicularity between 8.5° and 0.8°. Strictly speaking, this ERP’s plane is not the same as Listing’s plane because the “primary” direction given by vector $\vec{C_{r}F_{a}}$ is not perpendicular to it. Numerical simulations presented in [35] and in this study indicate that Listing’s law and the half-angle rule can only be approximately satisfied due to the ubiquitous misalignment of the healthy human eye’s optical components. They should be compared with experimental measurements of the Listing plane in the primary position and the displacement plane between the tertiary eye’s positions.

The above discussion of the perpendicularity of the line through the fixation point $F_{a}$ to the ERP frontal plane can be compared, although not directly, to the validity measurements of Listing’s law, as discussed in the next section.

### 5.2. Comparison of Simulations with Experimental Measurements

Testing Listing’s law requires precise measurements of ocular torsion. As mentioned earlier, the traditional method in [4,67] uses a scleral search coil in which 3D eye positions were measured in Fick coordinates and ocular torsion values were compared with theoretical values predicted by Listing’s law. An example of ocular torsion data from [67] is presented in the Table 2. A more direct method using a magnetic research coil with quaternion representations was presented in [5], which provided algorithms for eye position, eye velocity, primary position, and Listing’s plane extraction.

Data from many laboratories have shown that, in normal human subjects and rhesus monkeys, saccades and smooth-pursuit eye movements start and end in eye positions that approximately obey Listing’s law [4,5,6,67,68]. The simulated ocular torsions between the ERP’s fixation at $F_{a}$ and the tertiary position in [35] and here in Figure 5, Figure 6 and Figure 7 generally agree with the measurement results of healthy human eye torsion.

Comparing the Listing plane in the primary position between simulations and experiments is difficult. The main reason is that Listing’s law holds only approximately [4,67], so that the theoretical notion of the Listing plane in primary position does not correspond precisely to any real eye position [5]. Therefore, the Listing plane is determined experimentally as the best-fit plane through measured 3D eye-orientation vectors; its normal vector defines the experimentally inferred primary direction. On the other hand, the Listing plane in the binocular system with AEs, based on contemporary ophthalmology studies, is identified with the ERP planes, which, for each eye, equal the frontal head plane. This plane corresponds to the binocular eyes’ posture, with the eye muscles’ natural tonus resting position, which serves as a zero-reference level for convergence effort and for a unique abathic distance fixation (of about 1 m) of the empirical horopters, distinguished by a straight frontal line horopter; cf. Figure 2c.

The violation of Listing’s law for the binocular system with AEs was reported in [35]. The simulations shown in Figures 6 and 7 of that reference indicate that vertical eye shifts from ERP fixation at $F_{a}$ produce no change in eye torsion. However, the horizontal shifts produced small but non-zero torsion. More importantly, the iso-disparity curves were hyperbolas with the vertex close to the shifted fixation (for a 40 cm horizontal shift, the hyperbola’s vertex was only 20 cm away from the fixation point), which, through the coarse disparities, can affect our impression of immersion in the 3D ambient environment [69] despite receiving 2D projections on the retinas.

Another test of Listing’s law involves the reported standard deviation of scatter of measured eye orientation away from the best-fit Listing plane. It is given by the angular spread of how far the eye’s torsional orientation deviates from the ideal plane predicted by Listing’s law. The thickness of the Listing plane, which indicates the precision of Listing’s law, as reported in the literature, is as follows: [70] found a mean thickness of 1.5° for fixations, whereas [6] reported 1.4±0.5°. This is further discussed in Section 5.4 in relation to clinical data.

The experimental results of the half-angle rule can be compared to the simulations performed in this study. This is done by discussing the tilt-angle coefficient (TAC) for saccadic eye displacement. The eye angular-velocity axis tilt can be quantified by the TAC, the ratio of the angle of tilt of the eye-velocity axis to the angle of eye-position eccentricity; see [71,72,73,74] for examples. If a TAC is 0.5, the half-angle rule is precisely satisfied. Although I simulated the half-angle rule for displacements rather than velocities, the heuristic reason is as follows. The RV $r^{12}$ for each eye when fixation changes from $F^{1}$ to $F^{2}$ given in Equations (12) and (13) directly leads to the definition of the angular velocity for the corresponding eye given in Equation (47) in the moving eye frame and in Equation (49) in the ERP inertial frame that were obtained in [75]. Thus, the displacement RV $r^{12}$ well approximates the angular velocity for the saccadic straight-path movement. This will be confirmed by the experimental and simulation results presented in the Table 3.

**Figure 10:**
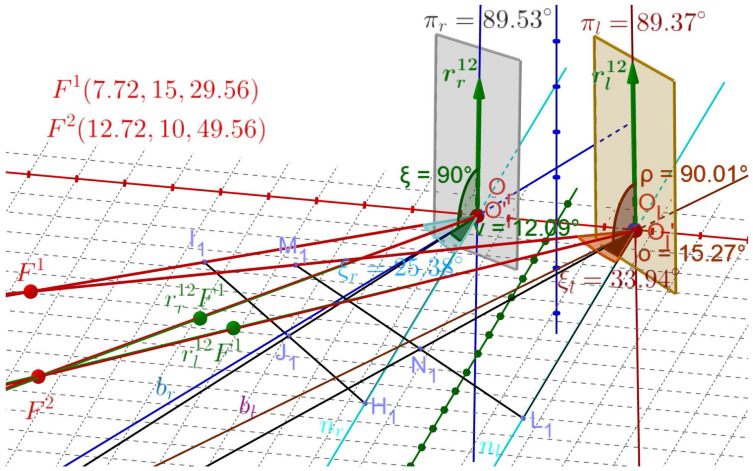
TAC for $F^{1}\to F^{2}$. For the right AE, two points $I_{1}$ and $H_{1}$ of the same distance from $O_{r}$ are selected on the visual axis through $F^{1}$ and the normal line $n_{r}$, respectively. The point $J_{1}$ is chosen on the segment $I_{1}H_{1}$ such that the angle between the normal line $n_{r}$ and the line containing $r_{r}^{12}$ and $J_{1}$ is as close to 90° as I could obtain. TAC is the ratio of the angles ν=12.09° between the line through $J_{1}$ and $n_{r}$ and $\xi_{r}=25.38{}^{\circ}$. The same is done for the left AE.

TAC for $F^{2}\to F^{1}$ is obtained along similar lines as explained in Figure 10.

We see in Table 3 that TAC values obtained in the study for oblique displacements are close to the averages of TAC values obtained for horizontal and vertical saccades. The novel derivation in [75] of the angular velocity directly from Rodrigues’ vectors rotating the eyes between tertiary postures, and simulations in Figure 8 and Figure 9 confirm a good approximation of straight saccadic movement by displacements between fixations.

### 5.3. Implications in Vision Science

It is well recognized in neurophysiological vision that the brain plays a clear role in coordinating the kinematics of visually guided eye movements. For instance, research suggests that the superior colliculus (SC) encodes the target’s location relative to the current gaze. Regardless of the eye’s starting position, the SC encodes the amplitude and direction (vector) required to reach the next position. These findings were first reported in nonhuman primates (cf. [78]) and subsequently confirmed in human subjects [79]. When moving from one tertiary position to another, the eyes typically take the most efficient path. This path is a rotation about a single axis that satisfies the half-angle rule and changes the direction of the fixation axis. Moving between tertiary positions induces ocular torsion relative to the head. The amount of torsion at each point in the movement is determined by its relationship to the primary position, ensuring the orientation remains consistent with Donders’ Law. Thus, the primary position should always be available as a reference during eye movement.

Further, the ubiquitous feature of the neural processing of intended eye movements is the efference copy (EC) of the oculomotor command [80]. The brain uses EC during pursuit eye movements to distinguish self-generated retinal image shifts from external movements, ensuring perceptual stability. It acts as a predictive feedforward mechanism, updating visual systems about eye position before sensory feedback arrives [81]. Also, during fast saccadic eye movements, the visual sensitivity is markedly reduced. Before the saccade is executed, the EC of oculomotor command is used to remap the receptive fields of the current fixation landmarks to the receptive fields of their future impending saccade’s target. This predictive remapping integrates objects’ features across fixations, maintaining the stability of perception [82].

Moreover, it has recently been recognized that the oculomotor plant also significantly controls the extraocular muscles acting on the eyeball via the pulley system. The prevailing model is based on the *Active Pulley Hypothesis*: the extraocular muscles have two layers: a global layer (GL) of EOM fibers that rotate the eye, and an orbital layer (OL) of fibers that insert onto the pulley sheath to actively position the eye. The pulley suspensions also contain smooth muscles, innervated by the autonomic nervous system, which may contribute to pulley positioning, particularly during focusing on a near object during convergence and during eccentric gaze to maintain a single, fused image [83,84,85,86,87]. This research also argued that these integrated actions help determine the direction of muscle pull and ensure precise, coordinated eye movements that conform to kinematic laws, such as Listing’s law and the related half-angle rule, to optimize ocular torsion and, hence, the noncommutativity of 3D eye rotations.

These aspects were experimentally tested in 2006 by [88] and in 2011 by [89], both for rhesus monkeys. The results indicated that although the brain plays a clear role in coordinating movement kinematics, the ocular plant significantly contributes to the peripheral mechanical optimization of the kinematic constraints necessary for visually guided eye movements. Nevertheless, as mentioned above, the eyes are constrained to fixate on a part of the viewed object, so that a binocular control system couples the 3D movements of the eyes, and a monocular torsion model should be generalized to the binocular case, including misaligned eyes’ optics. Consequently, the problem of the precise determination of the noncommutative behavior underlying the half-angle rule was never conclusively formulated.

In the study presented here, the decomposition of the change in eye posture into the torsion-free rotation with RV $r^{i}$ that gives the visual axes direction change and ocular torsion is represented by RV $q^{i}$ when, in both cases, the right and left eyes rotate from the ERP to $F^{i}$ (i=1,2) as geometrically constructed in Section 2.3. The RVs $r^{12}=-r^{21}$ when the fixation changes between tertiary bifixations $F^{1}$ and $F^{2}$ are expressed in terms of torsion-free RVs $r^{1}$ and $r^{2}$ of rotations from $F_{a}$ to $F^{i}$ (Equations (14) and (15)), which also holds in simpler form for $q^{12}=-q^{21}$ (Equations (22) and (23)).

I propose that the changes between tertiary bifixations $F^{1}$ and $F^{2}$ be referenced to the bifixations from ERP, $(r^{1}$, $q^{1})$ and $(r^{2}$, $q^{2})$ as expressed by Equations (14), (15), (22), and (23). It most likely involves an analog of EC discussed above: a rotation from $F_{a}$ to tertiary fixations. This hypothesis differs from the traditionally hypothesized series of so-called virtual rotations to and from the primary position between fixations, as originally proposed by Helmholtz [1] and later discussed by Hepp [12].

The motivation for my hypothesis that a neural analog of EC references fixations to the ERP is that, in this study, the primary position is defined by the eyes’ physiological resting position (see Section 3.1), rather than the standard ad hoc assumption of the primary position when both eyes are looking at infinity. This resting position is implied by misaligned optical components and is consistent with the eyes’ muscles’ natural tonus, corresponding to the abathic distance fixation for the empirical horopters. It can resolve the objection raised by Hess [16] regarding the above virtual motions, namely that they entail considerable computation and lack plausibility as a time-critical strategy for target tracking.

If my hypothesis is true, it can represent the physiological constraint that minimizes ocular torsion and enables the brain to track a sequence of fixations. Thus, the results of this study, supported by simulations, may point to Listing’s law and the half-angle rule as emergent properties of the geometry of kinematics. Classically, the amount of torsion at each point in the movement is the relationship between the angular velocity and the primary position, thereby ensuring the orientation remains consistent, as described by Donders’ law from which Listing’s law and its corollary, the half-angle rule, were derived. However, here the primary position is replaced by the ERP, corresponding to binocular fixation $F_{a}$. It is consistent with the remark raised in [15] that a binocular control system couples the 3D movements of the eyes and that an existing model of monocular torsion should be generalized to the binocular case of eye rotation with misaligned eyes’ optics. Thus, the precise relationship between the binocular formulation of Listing’s law and Donders’ monocular law should be carefully studied.

If the description proposed here, which seems to be supported by simulations, that Listing’s law arises naturally from the geometry of eyes’ kinematics is correct, then it should be upheld by the oculomotor plant. It would provide a significant cost-benefit. In any case, the pulley system must align with the misaligned optical components of the eyes, which vary among individuals but are conveniently represented by misalignment angles of AE. This seems to be important for precision control. Without properly functioning pulleys, strabismus (misalignment of the eyes) can occur, leading to double vision or disruption of depth perception, and the noncommutativity of the sequence of eye rotations can adversely affect world understanding, with unpleasant consequences.

### 5.4. Binocular Listing’s Law and Its Relevance to Clinical Practice

Listing’s law is undoubtedly genuine, even if approximate, the 3D eye-movement rule. However, its functional purpose, such as “diminish torsion”, “optimize muscles,” or “preserve clear binocular vision,” is not fully understood because, otherwise, cases of disease like strabismus and neural/muscular disorders should be clearly identified when Listing’s law fails.

The most relevant research papers addressing different aspects of strabismus and eye-movement disorders are listed in Table 4. In those studies, some questions remained unanswered: What are the effects of strabismus surgery on 3D eye orientation, or what are the consequences of surgery for torsion despite good eye realignment? Since the answers involve binocular Listing’s law, which was only precisely formulated in the study presented here. Thus, the results of this study should be included in those answers.

Nevertheless, the methodologies in the clinical studies listed in Table 4 do not allow direct comparison with the results reported in this paper, in which eyes are modeled using AE. The main reason is the differences among the eye’s visual, optical, and fixation axes and the lens optical axis. Thus, ocular torsion is best approximated as rotation about the lens’s optical axis rather than the visual axis, as commonly assumed in vision science. For example, in [94] listed in Table 4, ocular rotations are defined around the line of sight, which best agrees with the fixation axis but sometimes is identified with the visual axis.

Therefore, clinical studies should carefully use eye-movement recordings, especially torsional movements, with a non-invasive, high-resolution video oculography recently proposed in [65,66] and discussed in Section 4.1. In this recording, tracking the angular shift of the iris pattern about the pupil center is consistent with the definition of ocular torsion as given in Figure 4, but not with the ocular torsion defined around the line of gaze that most closely matches the fixation axis or the visual axis.

Moreover, in [94], the primary position is mathematically linked to Listing’s plane. Thus, Listing’s plane is best fitted from many experimental measurements, and the primary position is taken to be perpendicular to it. As discussed above, in the binocular system with AEs, where the Listing plane is substituted by the ERP, and the eye’s primary direction deviates from perpendicularity between 8.5° and 0.8° for 0°≤β≤4.5°; cf. Section 5.1. The study presented here substitutes the ERP for Listing’s plane with the primary direction deviating from perpendicularity as a function of eye misalignment of optical components. It is recommended to measure the angles of asymmetry, as defined by the AE model, before and after surgery to monitor their changes.

## 6. Conclusions and Future Study

In healthy human eyes, the fovea is displaced on the retina from the posterior pole, and the crystalline lens is tilted away from the eye’s optical axis. The classical Listing’s law, which optimizes ocular torsion by constraining the single eye’s redundant torsional degree of freedom, has overlooked the human eye’s misaligned optical components. Their inclusion in the Listing’s law and the related half-angle rule formulations discussed here has far-reaching consequences, which this article examined.

The ongoing work focuses on the geometric kinematics of bifixation in the eyes. It consists of formulating RVs directly in terms of the frame vectors used to specify AE orientation and of explicitly deriving the rotation matrix that accounts for the full change in the eyes’ posture, including ocular torsion. In the preliminary report in [75], this analytical formulation enables us to study how the angular velocity in the eye’s moving frame and the ERP initial (inertial) frame are related to the torsion-free and torsional parts of AE’s rotations. In particular, it proposed a new derivation of the angular velocity directly from the RV for the gaze changes between the tertiary eye’s positions.

## Figures and Tables

**Table 1:** Summary of the notation and important definitions used in the text. The subscripts ‘*r*’ and ‘*l*’ indicate the right and left eye.

AE	Asymmetric eye that models healthy human eye’s misaligned optical components
$C_{r}$, $C_{l}$	Eyes rotation centers
$N_{r}$, $N_{l}$	Eyes nodal points
$O_{r}$, $O_{l}$	Image plane optical centers
α, β, γ, ε	Angles of misalignment of optical components
$F_{a}$	Binocular fixation of the ERP distinguished by a straight frontal horopter
ERP	Eye resting posture for fixation $F_{a}$ that replaces the primary position in Listing’s law
$\left(i_{r},j_{r},k_{r}\right)$, $\left(i_{l},j_{l},k_{l}\right)$	Frames at $O_{r}$ and $O_{l}$ on the frontal plane of ERP that agree with the head frame
$\left(a_{r},b_{r},c_{r}\right)$, $\left(a_{l},b_{l},c_{l}\right)$	Rotated ERP frames on the image planes when $F_{a}$ changes to *F*
$\left(a_{r}^{\prime },b_{r}^{\prime },c_{r}^{\prime }\right)$, $\left(a_{l}^{\prime },b_{l}^{\prime },c_{l}^{\prime }\right)$	Rotated frames in the plane spanned by js and bs that align js and bs
$\phi_{r}=∠\left(j_{r},b_{r}\right)$, $\phi_{l}=∠\left(j_{l},b_{l}\right)$	Angles of rotation aligning js and bs vectors
$\tau_{r}=∠\left(k_{r},c_{r}^{\prime }\right)$, $\tau_{l}=∠\left(k_{l},c_{l}^{\prime }\right)$	Definition of eye’s torsion angles
$r_{r}=\tan\left(\phi_{r}/2\right)n_{r}$, $r_{l}=\tan\left(\phi_{l}/2\right)n_{l}$	Rodrigues’ vector (RV) rotating ϕ degrees around the line containing unit vectors $n_{r}$ and $n_{l}$
$r_{r}^{12}$, $r_{l}^{12}$	Torsion-free Rodrigues’ vectors when fixation changes between tertiary postures from $F^{1}$ to $F^{2}$
$q_{r}^{12}$, $q_{l}^{12}$	Torsional Rodrigues’ vectors when fixation changes between tertiary postures from $F^{1}$ to $F^{2}$
Binocular half-angle rule	If fixation changed between tertiary postures from $F^{1}$ to $F^{2}$, the Rodrigues’ vectors are in the plane that is tilted out of the ERP plane by half of the eccentricity of $F^{1}$
$\sigma_{r}^{k}=\left(r_{r}^{k},\;q_{r}^{k}\right)$, $\sigma_{l}^{k}=\left(r_{l}^{k},\;q_{l}^{k}\right)$	Points of configuration space: Listing’s law
$\sigma_{r}^{kl}=\left(r_{r}^{\mathrm{kl}},\;q_{r}^{\mathrm{kl}}\right)$, $\sigma_{l}^{kl}=\left(r_{l}^{\mathrm{kl}},\;q_{l}^{\mathrm{kl}}\right)$	Points of configuration space: half-angle rule

**Table 2:** Mean ocular torsion values during diagonal target trajectories between primary–tertiary and tertiary–tertiary positions.

Target Position	Steps Primary–Tertiary	Steps Tertiary–Tertiary
Nasal up	−4.11	−4.39
Temporal down	−2.38	−2.40
Primary	1.24	0.83
Temporal up up	6.38	7.00
Nasal down	3.22	3.50
Primary	1.03	0.79

^1^ Ocular torsion values in degrees (+ = extorsion; − = intorsion).

**Table 3:** The experimental and numerical determination of TAC for the half-angle rule.

Study	Horizontal Saccade TAC	Vertical Saccade TAC	Oblique Displacement TAC
Tweed and Vilis 1990 [5]	0.5	-	-
Bruno and Van den Berg 1997 [76]	0.42	0.28	-
Crane et al. [73]	0.50	0.45	-
Thurtell et al. [74]	0.57	0.30	-
Thurtell et al. [77]	0.57	0.34	-
Current ^1^			
$F^{1}\to F^{2}$			
Right Eye	-	-	0.48
Left Eye	-	-	0.45
$F^{2}\to F^{1}$			
Right Eye	-	-	0.46
Left Eye	-	-	0.46

^1^ Data is from simulations explained in Figure 10.

**Table 4:** Important reviews/papers that directly connect Listing’s law and half-angle rule with torsional deviation, binocular alignment, and strabismus.

Study	Clinical Issues Addressed
Wong. 2004 [90]	This review explained Listing’s law, Listing’s plane, torsion, ocular motor palsies, and why the law matters clinically for binocular vision and strabismus.
Melis et al. 1997 [18]	Direct strabismus study. Using dual magnetic search coils found in a stereoblind strabismic patient, each eye could have a well-defined Listing plane, but the plane depended on which eye was fixating.
Bosman et al. 2002 [91]	This study provided evidence that strabismic eye surgery can change adherence to Listing’s law and the relative orientation of the two eyes’ planes.
Wong et al. 2002 [92]	Fourth nerve palsy is the cyclovertical strabismus condition. This study found that acute fourth-nerve palsy can violate Listing’s law, whereas chronic palsy may obey it, suggesting neural adaptation.
Wong et al. 2002 [93]	Sixth nerve palsy causes horizontal strabismus, often esotropia from weak lateral rectus. This paper compares horizontal motor palsy with cyclovertical palsy.
Straumann et al. 2003 [94]	Trochlear nerve palsy affects the superior oblique muscle and produces vertical/torsional strabismus. This paper studies how primary position and Listing’s law differ in acquired versus congenital trochlear nerve palsy.
Fesharaki et al. 2008 [95]	Skew deviation is a vertical strabismus with abnormal ocular torsion. This paper directly asks whether patients with skew deviation obey Listing’s law and reports acute-versus-chronic adaptation effects.
Demer. 2007 [96]	This paper studied pulley pathology in incomitant strabismus. The results are consistent with mechanical rather than central neural implementation of Listing’s law.
Miller. 2007 [97]	This paper discussed how coordinated pulleys account for half-angle behavior and how pulley heterotopy can cause incomitant strabismus.

## Data Availability

Data generated in *GeoGebra* will be of little help without accompanying instructions, as it involves more than 100 ready-to-use geometric tools, with half of them hidden, cf. Section 3.3. Alternatively, the *GeoGebra* manual can be downloaded online from https://geogebra.github.io/docs/manual/en/ (accessed on 10 May 2026). The links to the simulations are available, along with individual instructions from the author, upon reasonable request.

## Abbreviations

The following abbreviations are used in this manuscript:

AE	Asymmetric eye
SE	Symmetric eye
ERP	Eye-resting posture that replaces the Listing plane and primary direction
VMC	Vieth–Müller circle
RV	Rodrigues’ vector
CS	Configuration space
SC	Superior colliculus
EC	Eferrence copy
OL	Orbital layer
GL	Global layer
EOM	Extraocular muscles
